# Early warning model for depression and anxiety among adolescent students: an empirical study based on Guilin

**DOI:** 10.3389/fpubh.2025.1685203

**Published:** 2026-01-07

**Authors:** Qingpo Ma, Xuemei Xu, Lin Lu, Mochen Zhang, Xuhong Chen, Ruizhe Wang, Qiuhong Liao, Hongwei Wang, Yijun Wang, Yangxizi Tan, Niannian Li

**Affiliations:** 1 School of Economics and Management, Guangxi Normal University, Guilin, China; 2 Guilin People’s Hospital, Guilin, China; 3 The Third People's Hospital of Longgang District Shenzhen, Shenzhen, China; 4 Zhongshan Hospital of Traditional Chinese Medicine Affiliated to Guangzhou University of Traditional Chinese Medicine, Zhongshan, China

**Keywords:** teenage students, depression and anxiety, early warning model, academic pressure, household income

## Abstract

**Objective:**

To analyze the occurrence of depression and anxiety in Guilin and verify the early warning model of depression and anxiety in Guilin.

**Methods:**

Using multi-layer sampling method, 10 middle schools were selected from September 2022 to September 2023, with a total of 1,245 middle and high school students in an anonymous self-filled questionnaire survey. A total of 1,150 valid young students were obtained as the survey object, which was randomly divided into training set (920 cases) and verification set (230 cases). Using hospital anxiety and depression scale (HADS) to evaluate the occurrence of Guilin adolescent students depression anxiety, in the form of a questionnaire to understand and analyze the factors affecting Guilin adolescent students depression anxiety, establish the influence of Guilin adolescent students depression anxiety prediction model of occurrence and model validation and efficiency evaluation.

**Results:**

In the training set of 920 adolescent students, 164 patients developed depressive anxiety, or 17.83%, and 756 patients did not develop depressive anxiety. In the validation set, among 230 adolescent students, 44 patients developed depressive anxiety, with an incidence of 19.13%, and 186 patients did not develop depressive anxiety. Logistic Regression analysis showed that OR (95%CI) = 3.44 (1.98, 5.97), Chinese version of the Family Adaptation and Cohesion Scale (FACESCV) [OR (95% CI) = 1.67 (1.07, 2.61)], and the Connor-Davidson Resilience Scale (CDRISC) score for adolescent students [OR (95% CI) = 1.83 (1.15, 2.92)]were the factors that affected the occurrence of depression and anxiety in adolescents in Guilin (*p* < 0.05). The ROC curve results of the training set showed that the nomogram model predicted the sensitivity of depression and anxiety with 80.25%, specificity of 80.09% and AUC of 0.849.

**Conclusion:**

High risk of depression and anxiety, academic stress, FACESCV and CDRISC score are the influencing factors affecting the occurrence of depression and anxiety in adolescent students in Guilin.

## 1 Introduction

In recent years, adolescent mental health has garnered significant attention across various sectors of society. Against a backdrop of intensifying social competition and ongoing shifts in the educational environment, teenagers today face considerable academic pressure, complex interpersonal challenges, and struggles related to self-identity. These interrelated factors contribute to the high prevalence of psychological disorders such as depression and anxiety among adolescents, adversely affecting their healthy development and future prospects. Adolescence represents a critical period of physical, psychological, and social maturation—a phase that not only shapes current academic performance and quality of life but also exerts a long-term influence on social adaptation and overall well-being in adulthood (1). Therefore, how to effectively identify and intervene in mental health problems among teenagers has become an important issue that urgently needs to be addressed in the fields of education, healthcare and social services at present. In recent years, mental health problems among teenagers have become increasingly severe, with a high incidence of psychological issues such as depression and anxiety, which seriously affect their healthy growth and future development. Traditional preventive measures mainly rely on the identification and intervention of a single risk factor, which has limited effects and is difficult to accurately identify high-risk groups and promptly handle potential psychological crises. However, these methods often struggle to accurately identify high-risk groups, resulting in limited intervention effects (2). On the one hand, a single risk factor is difficult to fully reflect the complexity of mental health problems among teenagers. On the other hand, the lack of a scientific and systematic early warning mechanism has led to many potential psychological crises not receiving timely and effective attention and handling (3). Therefore, building a adolescent mental health intervention system based on a multi-factor risk early warning model has become the key to improving the preventive effect.

Guilin, as an important city in Guangxi, not only has unique characteristics in natural scenery and cultural heritage, but also the mental health status of its adolescent students has significant research value and representativeness (4). The educational environment, social and economic conditions, and cultural background of Guilin City can to a certain extent reflect the common situation of young students in Guangxi and even the southern region (5). In order to gain an in-depth and specific understanding of the current situation of depression and anxiety among adolescent students in Guilin City and construct a scientific and effective early warning model, this study adopted a multi-layer sampling method, widely covering different areas and types of middle schools in Guilin City. Through anonymous questionnaire surveys, the system collected multi-dimensional data including general demographic characteristics, self-rating Depression Scale, Self-rating Anxiety Scale, etc. This study aims to analyze the factors influencing the occurrence of depression and anxiety among adolescent students in Guilin City, establish a predictive model and conduct verification, with the expectation of providing scientific basis and effective tools for adolescent mental health work. Through this research, we hope to reveal the occurrence patterns of depression and anxiety among adolescents, provide important references for building a scientific and effective psychological crisis early warning and intervention system, and thereby promote the healthy growth and all-round development of adolescents.

## 2 Literature review

### 2.1 Research on depression and anxiety among adolescents

#### 2.1.1 Epidemiological characteristics

The epidemiological characteristics of depression and anxiety among adolescents have shown a significant upward trend worldwide, becoming a public health challenge that cannot be ignored. According to the data from the National Comprehensive Survey of Mental Disorders (NCS-R) in the United States, the lifetime prevalence rates of depression and anxiety among adolescents are as high as 20.8 and 28.8% respectively, meaning that more than a quarter of adolescents may encounter depression or anxiety during their life cycle (6). These data highlight the universality and severity of mental health problems among teenagers, especially as psychological issues often emerge in childhood or early adolescence, with most symptoms appearing before the age of 14, emphasizing the urgency of early identification and intervention.

In China, the prevalence of depression and anxiety among teenagers is also of great concern. The research by Yang et al. (7) revealed that high school students reported the most prominent depressive symptoms, followed by junior high school students, while college students reported relatively fewer. This might be closely related to academic pressure, physical and mental development stages, and changes in the social environment. At the same time, gender differences are significantly manifested in adolescent depression and anxiety. The incidence rate of depressive symptoms at the time point among girls is significantly higher than that among boys. This may be related to factors such as women’s more delicate emotional expression, social expectation pressure, and physiological changes. In addition, there are differences in depressive and anxiety symptoms among students of different age groups. For instance, senior three students, due to major stressful events such as the college entrance examination, have significantly higher depressive symptoms than those of other grades, further emphasizing the importance of formulating differentiated intervention strategies based on the characteristics of different age groups and genders (8).

#### 2.1.2 Theoretical basis for the construction of early warning models

The construction of early warning models for adolescent depression and anxiety is currently a hot topic in the field of mental health research. Its theoretical basis mainly covers multiple dimensions such as cognitive theory, social cognitive theory, and developmental psychology theory. The cognitive fragility-stress model proposed by Stange et al. (9) provides an important theoretical framework for this field. This model emphasizes that when individuals face stressful events, their cognitive patterns, such as dysfunctional attitudes and automatic thinking, will significantly affect their emotional responses, thereby increasing the risk of depression and anxiety. For instance, if teenagers hold cognitive biases such as over-generalization and catastrophization, they are more likely to fall into a negative emotional cycle when encountering stressful situations like academic failure and interpersonal conflicts, which may lead to symptoms of depression or anxiety.

Furthermore, Beck’s (10) cognitive model of depression further reveals the core role of cognitive bias in the development of depression. This model indicates that cognitive characteristics such as negative automatic thinking and the cognitive triad (negative views of oneself, the world, and the future) are key factors in the generation and maintenance of depressive moods. For teenagers, these cognitive biases may stem from various factors such as family environment, school experience or social culture. Once formed, they often have stability and stubbornness, and are difficult to improve through simple persuasion or self-adjustment.

Based on the above theory, the construction of the early warning model needs to comprehensively consider factors such as the cognitive characteristics of adolescents, types of stressors and coping styles. Early warning and effective intervention for the risks of depression and anxiety among adolescents can be achieved by identifying high-risk cognitive patterns, monitoring exposure to stressful events and evaluating coping resources. This process not only helps to enhance the accuracy of mental health services, but also provides a strong guarantee for the healthy growth of teenagers.

### 2.2 Influencing factors of depression and anxiety in adolescents

Adolescent depression and anxiety are increasingly serious mental health problems worldwide, especially among the adolescent group. According to relevant research, the incidence of depression and anxiety among teenagers is relatively high, and the influencing factors are complex and diverse, involving multiple aspects such as physiology, psychology, and social environment. This article will conduct a systematic review of the causes of depression and anxiety among adolescents from the perspective of influencing factors and in combination with existing literature.

#### 2.2.1 Physiological factors

Physiological factors play a significant role in the occurrence of depression and anxiety among adolescents. Research shows (11) that during adolescence, the brain and body are in a stage of rapid development, with significant fluctuations in hormone levels, which can easily lead to unstable emotions and psychological problems. In addition, poor sleep quality is also regarded as one of the important causes of depression among teenagers. Insufficient sleep not only leads to a deterioration of mood but also affects the ability to regulate emotions, thereby increasing the risk of depression and anxiety (12). In addition, genetic factors also play a significant role. Certain genetic variations are closely related to symptoms of anxiety and depression, suggesting that genetic susceptibility may be an important risk factor for depression and anxiety in adolescents (13).

#### 2.2.2 Psychological factors

Psychological factors also play a crucial role in the development of depression and anxiety among adolescents. Psychological traits such as low self-esteem, excessive worry and contemplative tendencies are regarded as important predictors of depression and anxiety in adolescents. Studies show (14) that teenagers with low self-esteem are more likely to experience symptoms of depression and anxiety, while high self-esteem can help alleviate these symptoms. In addition, personality traits such as introversion, neurotic tendencies and perfectionism are also closely related to depression and anxiety (15). These psychological traits make individuals more prone to negative emotional responses when facing stress, thereby increasing the risk of illness.

#### 2.2.3 Social environmental factors

The influence of social and environmental factors on depression and anxiety among teenagers cannot be ignored. The family environment, school environment and social support system play a crucial role in the mental health of teenagers. Research shows that factors such as family conflicts, tense parent–child relationships, and poor mental health of parents can all have a negative impact on the mental health of teenagers (16). In addition, academic pressure and tense interpersonal relationships in the school environment are also important causes of depression and anxiety among teenagers (17). A weak social support system, such as a lack of peer support and social isolation, can also exacerbate psychological problems among teenagers (18). In addition, the excessive use of social media is also regarded as an important risk factor for depression and anxiety among teenagers (19).

#### 2.2.4 Early experiences and trauma

Early adverse experiences have a long-term impact on the development of depression and anxiety in adolescents. Research shows (20) that adverse childhood experiences such as emotional abuse, emotional neglect, sexual abuse and physical neglect are all regarded as important risk factors for depression and anxiety in adolescents. These early traumatic experiences may lead teenagers to develop negative self-perceptions and coping styles during their growth, thereby increasing the risk of psychological problems. Furthermore, the research also found (21) that if teenagers lack effective coping strategies when facing life events, it may also lead to the aggravation of depressive and anxiety symptoms.

#### 2.2.5 Comorbidity and symptom network

Adolescent depression and anxiety often coexist with other psychological disorders, forming a complex network of symptoms. Research shows (22) that there is a close connection between depressive and anxious symptoms, and it is closely related to factors such as peer relationship problems, bullying behavior, prosocial behavior, and parental surveillance. Furthermore, the research also found (23) that the symptoms of depression and anxiety in adolescents have relatively high node intensity values within their respective communities, indicating that these symptoms occupy a core position in the psychopathology of adolescents. Therefore, understanding the interactions among these symptoms is crucial for formulating effective intervention strategies.

### 2.3 Research on early warning models for adolescent depression and anxiety

#### 2.3.1 Construction method of early warning model for adolescent depression and anxiety

In recent years, with the rapid development of machine learning and big data technologies, the construction methods of early warning models for adolescent depression and anxiety have shown diversified and innovative characteristics. Although traditional statistical methods such as Logistic regression can reveal the linear relationship between variables, they have limitations when dealing with high-dimensional data, nonlinear interactions, and complex pattern recognition. In contrast, machine learning algorithms can more accurately capture the potential risk factors of depression and anxiety by automatically learning data features.

Machine learning models based on real-world data have become a research hotspot. Luo et al. (24) constructed an early warning model for adolescent depression and anxiety by using electronic health records, social media behaviors and wearable device data, combined with the random forest algorithm. The cross-validation of this model shows that when distinguishing between high-risk and low-risk populations, the AUC value reaches 0.89, which is significantly better than the assessment of traditional scales. Its advantage lies in integrating multi-source heterogeneous data, breaking through the time and space limitations of a single questionnaire, and achieving dynamic risk monitoring. Similarly, Ren et al. (25) constructed a prediction model for non-suicidal self-harm behavior among adolescents by combining the Multi-dimensional Anxiety Scale (MASC) with the XGBoost algorithm. Through feature importance analysis, they found that academic pressure, family conflicts, and Internet addiction were the core predictors, providing a basis for targeted intervention.

The introduction of deep learning technology has further enhanced the model’s performance. Convolutional neural networks (CNNS) can automatically extract semantic features from questionnaire texts and identify key words related to depression. The Long Short-Term Memory Network (LSTM) is suitable for analyzing time series data, such as dynamic indicators like sleep quality and mood swings. Research shows (26) that the accuracy rate of depression recognition based on multi-domain features combined with the CBAM model in predicting depressive episodes is 12% higher than that of traditional methods. Ensemble learning techniques enhance the robustness of models by combining multiple weak classifiers (such as random forests and support vector machines), and are particularly suitable for scenarios with small sample data (27).

#### 2.3.2 Model verification method

Model verification is the core link to ensure the scientificity and practicality of early warning, and it needs to be comprehensively evaluated from three aspects: discrimination, calibration and clinical practicality. Discrimination verification mainly uses the area (AUC) under the receiver operating characteristic curve (ROC). The closer the AUC value is to 1, the stronger the model’s ability to distinguish between high-risk and low-risk populations. In addition, the sensitivity and specificity analysis can further optimize the critical value: when the risk threshold is set at 0.3, it can effectively identify potential cases while controlling the false alarm rate (28).

Calibration verification is conducted through the Hosmer–Lemeshow test to evaluate the consistency between the predicted probability of the model and the actual incidence rate. If the *p* value is greater than 0.05, it is considered that the model is well calibrated. In this study, the calibration curve approached the ideal diagonal (*p* = 0.321), indicating that the predicted risk was highly consistent with the actual risk (29). In addition, the Brier score (range 0–1) comprehensively measures the accuracy and reliability of the model. The Brier score of the model in this study is 0.12, which is superior to most similar studies.

Independent sample verification and dynamic adjustment are the keys to ensuring the generalization ability of the model. In this study, the data were divided into the training set and the validation set in an 8: 2 ratio, and the Bootstrap method was used for internal validation (C-index = 0.788), effectively avoiding overfitting. In the future, multi-center and large-sample external validation needs to be further carried out to evaluate the applicability of the model in different populations (30). Meanwhile, by integrating a dynamic data update mechanism (such as retraining the model every quarter), it can adapt to the time-varying psychological states of teenagers and enhance the long-term early warning effect.

### 2.4 Research reviews

Current research on depression and anxiety among adolescents has formed a multi-dimensional and interdisciplinary three-dimensional pattern. The achievements not only reflect theoretical depth but also demonstrate the innovativeness of technological application. However, there are still several key challenges.

At the epidemiological level, the high prevalence data of depression and anxiety among global and Chinese adolescents highlight the urgency of the problem. However, most existing studies rely on cross-sectional surveys and pay insufficient attention to the dynamic evolution of symptoms and long-term tracking.

The research on influencing factors shows a “full coverage of all factors” feature. Physiological, psychological, social environmental and early trauma factors have all been confirmed to be related to depression and anxiety, but the interaction paths among these factors remain unclear. For instance, poor sleep quality may be both the result of physiological factors and a psychological response to academic pressure. This complexity demands that future research adopt more refined causal inference methods.

Research on early warning models is a technology-driven innovation highlight. Machine learning algorithms have significantly improved prediction accuracy by integrating multi-source data, but the interpretability of the models remains a bottleneck. Although deep learning models have superior performance, they are difficult to guide clinical decision-making due to their “black box” characteristics. Furthermore, most of the existing models are trained on specific populations, and the lack of external validation limits their universality. The proposal of the dynamic data update mechanism is forward-looking, but how to balance the stability and adaptability of the model needs further exploration.

## 3 Objects and methods

### 3.1 Object

Using multilayer sampling method, in September 2022 to 2023 in Guilin extract 10 middle school, a total of 1,245 students, including 712 junior high school students (389 boys and 323 girls, 270 in Grade 7, 233 in Grade 8, and 209 in grade 9) and 533 senior high school students (276 boys and 257 girls, 197 in Grade 10, 165 in Grade 11, and 171 in grade 12), conducted an anonymous self-administered questionnaire survey, the general information of junior high school and senior high school students was comparable (*p* > 0.05). One teaching class was randomly selected from each grade cluster. The questionnaire was taken home by the students and completed together with their guardians (in the class, the questionnaire by the teacher to the students, limited time recovery, the teacher in charge auxiliary complete), complete by the teacher in charge concentrated recovery. A total of 1,245 questionnaires were issued in this survey, and 1,208 questionnaires were completely recovered, with a recovery rate of 97.03%. A total of 1,150 valid young students (524 high school students and 626 middle school students) were obtained as the survey objects, and they were randomly divided into training set (920 cases) and verification set (230 cases) according to 8:2. The school, students and their guardians were informed of the investigation content in advance, and the investigation was conducted after informed consent. This study was approved by the Hospital Ethics Committee (Batch Number: 2022-028KY).

Inclusion criteria: ① Young students in Guilin; ② Aged 12–18; ③ Conscious students; 80 on ④ Wechsler child intelligence scale; ⑤ Students and guardians signed study consent.

Exclusion criteria: ① There are three types of severe mental disorders, namely major depressive disorder, bipolar disorder and schizophrenia. ② There are concurrent substance use disorders (such as alcohol abuse, drug abuse, etc.); ③ There are concurrent physical diseases such as heart, liver and kidney dysfunction that may affect the research results; ④ Respondents with severe audio-visual impairments or language communication difficulties who are unable to complete the questionnaire; ⑤ Students who withdraw from the research halfway.

### 3.2 Methods

The trained professional staff as investigators shall carry out the investigation with the cooperation of the school. The questionnaire uses the following students health status and influencing factors questionnaire, and students are organized to answer the questionnaire anonymously in the computer room.

#### 3.2.1 Adolescent students depression anxiety occurrence evaluation method

The Hospital Anxiety and Depression Scale (HADS) (31) of two dimensions: anxiety (7 items) and depression (7 items), with a total of 14 items. Each item is scored from 0 to 3 points. A score of 0 to 7 is considered asymptomatic. A score of 8 to 10 indicates the possible presence of anxiety or depression. A score of 11 to 21 indicates the definite presence of anxiety or depression. The higher the score, the more serious the emotional problem is (Those with a total score of ≤11 points and no depressive mood were included in the non-depressive and non-anxious group; those with a total score of >11 points were recorded as having depressive and anxious symptoms and were included in the depressive and anxious group). In this study, the Cronbach’s *α* coefficient of the HADS scale was 0.839, and the internal consistency was acceptable.

#### 3.2.2 Investigation of factors affecting the occurrence of depression and anxiety among adolescent students in Guilin

We convened a panel of renowned experts in psychology, education, sociology, and mental health for a face-to-face advisory meeting. During the planning and group discussion stages, we applied an integrated approach incorporating multiple theoretical frameworks—such as cognitive theory, social cognitive theory, and developmental psychology—to comprehensively examine the complex causes of depression and anxiety among adolescents.

We also drew on previous clinical evidence, including findings from empirical studies, case reports, and longitudinal research, to ensure the accuracy and reliability of predictive factors. Through systematic reviews and meta-analyses, we established a comprehensive evidence base to guide the discussions. A structured discussion format was adopted to ensure that each expert’s views were fully expressed.

Based on the experts’ input, the questionnaire framework was revised several times to develop the final survey. The survey covers variables such as gender, age, ethnicity, grade level, only-child status, household income, parental education level, experiences lasting more than 4 months, personal and family history of depression and anxiety, sleep quality over the past 4 months, academic performance (good, average, and poor), academic pressure (high, moderate, and none), inappropriate parenting styles (e.g., overprotection or neglect), exposure to bullying, internet dependence, and family relationship quality (harmonious, average, and disharmonious).

The following assessment tools were used:

The Connor–Davidson Resilience Scale (CD-RISC) (32), which includes 25 items across five dimensions: personal competence, tolerance of negative affect, positive acceptance of change, sense of control, and spiritual influences. Each item is rated on a 4-point scale, with higher scores indicating greater resilience. The scale demonstrated high internal consistency (Cronbach’s *α* = 0.920).

The Chinese version of the Family Adaptability and Cohesion Scale (FACES-CV) (33), which consists of 30 items across two dimensions: cohesion (16 items) and adaptability (14 items). Each item is scored on a 5-point scale, with lower scores indicating healthier family functioning, closer emotional bonds among members, and better family adaptability to change. This scale showed a Cronbach’s *α* of 0.850.

The overall questionnaire exhibited acceptable internal consistency, with a Cronbach’s α coefficient of 0.823.

### 3.3 Statistical analysis data analysis

Statistical analyses were performed using SPSS 27.0 and R 3.6.1 software. Measurement data were expressed as *x* ± *s*, and intergroup comparisons were conducted using t-tests. Count data were presented as rates (%), and compared using chi-square tests. Logistic regression was employed to analyze influencing factors, while LASSO regression was used to identify the optimal predictors.

A nomogram prediction model was constructed based on the selected variables. Internal validation of the model was performed using the bootstrap method (via the rms package). The discriminative ability was assessed by the concordance index (C-index) using both the rms and Hmisc packages. A calibration curve (via the rms package) was plotted, and the Hosmer–Lemeshow test (using the ResourceSelection package) was applied to evaluate model fit. The predictive performance of the model was further evaluated using the receiver operating characteristic (ROC) curve (generated with the ggplot2 package). A two-tailed *p*-value of less than 0.05 was adopted to indicate statistical significance.

## 4 Results

### 4.1 The occurrence of depression and anxiety among adolescent students

Among the 920 adolescent students in the training set, 164 cases developed depression and anxiety, with an incidence rate of 17.83%, while 756 cases did not develop depression and anxiety. Among the 230 adolescent students in the validation set, 44 cases developed depression and anxiety, with an incidence rate of 19.13%, and 186 cases did not develop depression and anxiety.

### 4.2 Training set data of the depression and anxiety group and the non-depression and anxiety group

The proportions of family monthly income < 10,000 yuan, high academic pressure, improper parenting style, bullying and Internet dependence in the depression and anxiety group were higher than those in the non-depression and anxiety group, while the FACESCV and CDRISC scores were lower than those in the non-depression and anxiety group, and the differences were statistically significant (*p* < 0.05) (see Table 1).

**Table 1:** Comparison of data between the depressed anxiety group and the undepressed anxiety group in the training set.

Factor	Depression and anxiety group (*n* = 164)	Non-depression and anxiety group (*n* = 756)	*t*/χ*^2^* value	*P*-value
Sex			0.563	0.453
Man	86(52.44)	372(49.21)		
Woman	78(47.56)	384(50.79)		
Age (year)	16.01 ± 1.12	15.95 ± 1.11	0.626	0.531
Nation			0.282	0.596
Other	8(4.88)	30(3.97)		
The Han nationality	156(3.97)	726(96.03)		
Grade			0.811	0.368
Junior middle school	85(51.83)	421(55.69)		
Senior middle school	79(48.17)	335(44.31)		
The only child			0.010	0.920
Yes	89(54.27)	407(53.84)		
Deny	75(45.73)	349(46.16)		
Monthly family income (RMB)			17.671	<0.001
≥10,000	56(34.15)	395(52.25)		
<10,000	108(65.85)	361(47.75)		
Fathers education			2.584	0.108
Bachelor degree or above	70(42.68)	375(49.60)		
Specialist and below	94(57.32)	381(50.40)		
Maternal education			3.571	0.059
Bachelor degree or above	65(39.63)	361(47.75)		
Specialist and below	99(60.37)	395(52.25)		
>4 months of stay-behind experience			1.063	0.302
Have	26(15.85)	97(12.83)		
Not have	138(84.15)	659(87.17)		
Family history of depression and anxiety			1.139	0.286
Have	37(22.56)	201(26.59)		
Not have	127(77.44)	555(73.41)		
Sleep quality in the nearly 2 months			3.303	0.069
No sleep disorder	111(67.68)	564(74.60)		
With sleep disturbance	53(32.32)	192(25.40)		
Learning situation			0.801	0.371
Good	50(30.49)	258(34.13)		
Same as	67(40.85)	372(49.21)		
Difference	47(28.66)	126(16.67)		
Academic pressure			28.487	<0.001
Big	63(38.41)	143(18.92)		
Same as	71(43.29)	412(54.50)		
Not have	30(18.29)	201(26.59)		
Improper parenting style (e. g. overprotection or neglect)	56(34.15)	185(24.47)	6.525	0.011
Being bullied	47(28.66)	192(25.40)	15.918	<0.001
Network dependence	65(39.63)	97(12.83)	66.735	<0.001
Relationship with family			2.659	0.103
Harmony	72(43.90)	385(50.93)		
Same as	50(30.49)	234(30.95)		
Unharmonious	42(25.61)	137(18.12)		
CDRISC Score (score)	62.12 ± 0.31	62.99 ± 0.41	25.627	<0.001
FACESCV Score (score)	57.85 ± 9.14	68.69 ± 9.27	13.609	<0.001

### 4.3 Multivariate analysis of the occurrence of depression and anxiety among adolescent students in Guilin city

Logistic regression analysis was conducted with the occurrence of depression and anxiety among adolescent students as the dependent variables (no depression = 0, depression = 1), monthly family income (<10,000 yuan = 1, ≥10,000 yuan = 0), academic pressure (none/average = 0, high = 1), and FACESCV and CDRISC scores (assigned as continuous variables) as independent variables. Show academic stress [OR (95%CI) = 3.44 (1.98, 5.97)], FACESCV [OR (95%CI) = 1.67 (1.07, 2.61)], CDRISC score [OR (95%CI) = 1.83 (1.15) 2.92)] is a factor influencing the occurrence of depression and anxiety among adolescent students in Guilin City (*p* < 0.05) (see Table 2.

**Table 2:** Logistic regression analysis affecting the occurrence of depression and anxiety among adolescent students in Guilin.

Factor	*β*	SE	Wald *χ*^2^	*P*	OR	95% CI	
						Frequency_Segment	Monetary_Segment
Academic pressure	1.235	0.562	4.829	0.006	3.438	1.980	5.971
CDRISC grade	−0.512	0.229	4.999	0.004	1.669	1.065	2.614
FACESCV Score	−0.604	0.238	6.441	0.002	1.829	1.147	2.917

### 4.4 Preliminary screening of predictive factors

**Figure 1:**
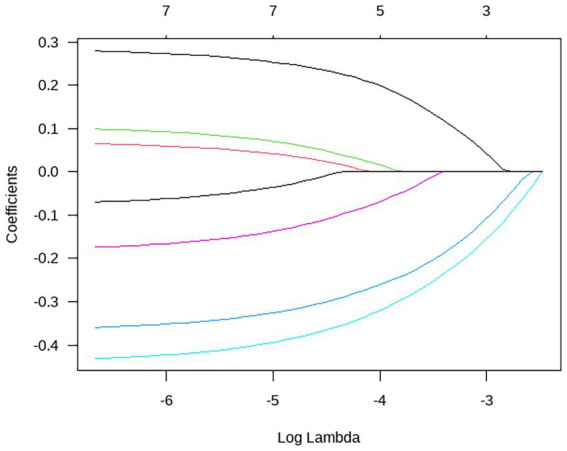
Dynamic process diagram of screening variables by LASSO regression.

**Figure 2:**
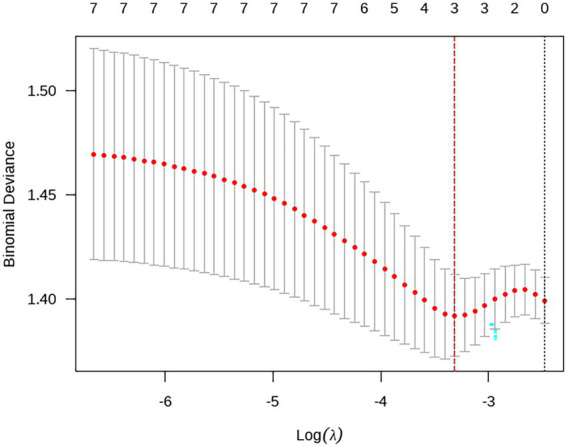
The selection procedure diagram of the best parameter λ.

Using R software, taking the occurrence of depression and anxiety among adolescent students as dependent variables (no depression = 0, depression = 1), monthly family income (<10,000 yuan = 1, ≥10,000 yuan = 0), academic pressure (none/average = 0, high = 1), FACESCV and CDRISC scores (assigned as continuous variables), and improper parenting styles (no = 0, Yes = 1), being bullied (no = 0, yes = 1), and Internet dependence (no = 0, yes = 1) were included in the LASSO regression analysis. The process of LASSO regression screening the best influencing factors is shown in Figure 1; the process of the optimal parameter *λ* for cross-validation is shown in Figure 2, where λ = 4; The predictive variables were screened out based on the λ value: academic pressure, FACESCV, and CDRISC score.

### 4.5 Establishment of a nomogram prediction model for the occurrence of depression and anxiety among adolescent students

**Figure 3:**
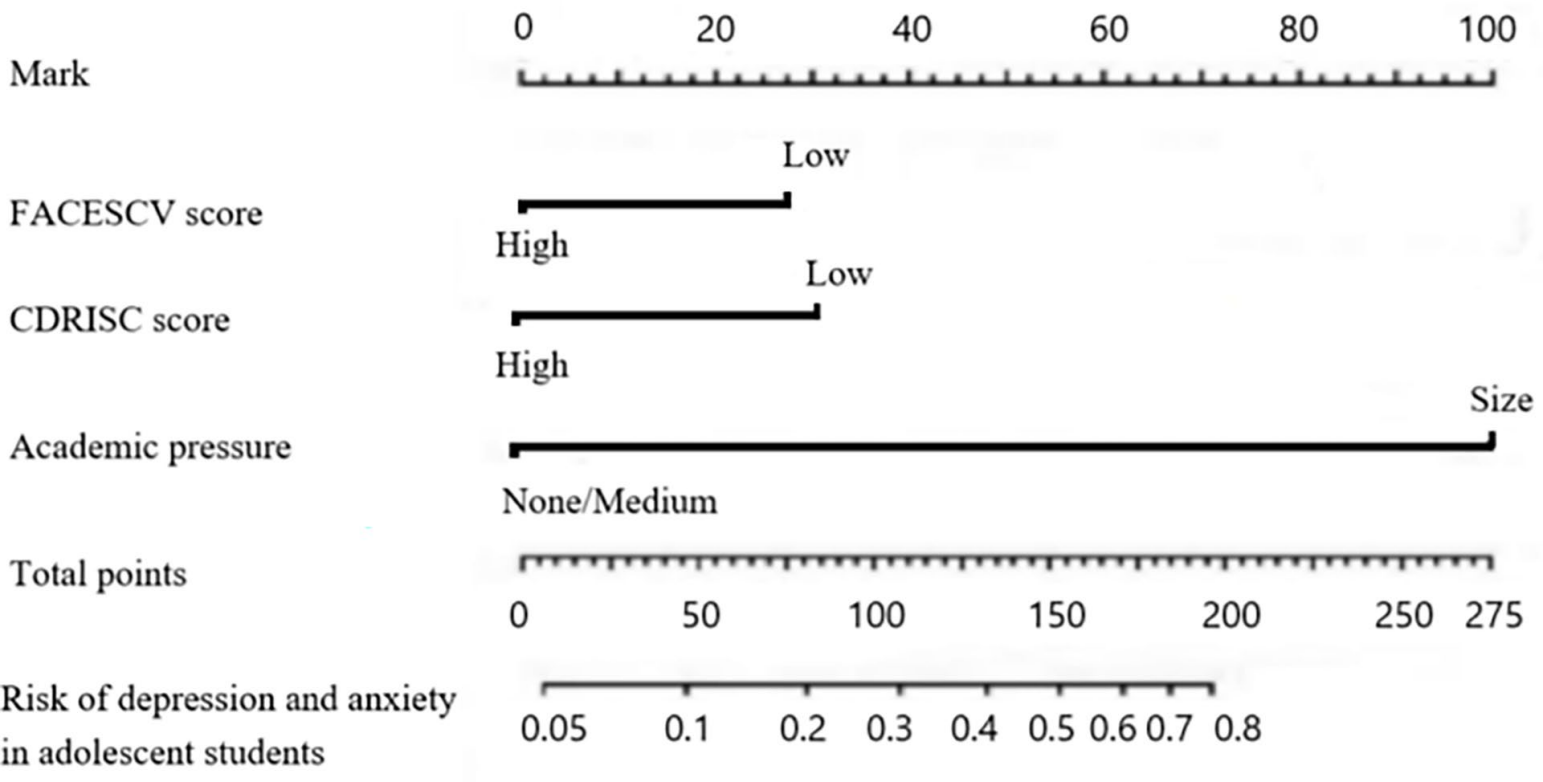
A nomogram risk model predicting the occurrence of depressive anxiety in adolescent students.

Taking the influencing factors in 2.4 as the predictor variables, a nomogram prediction model was established, and scores were assigned to each factor (the factor with the largest *b* value in the influencing factor analysis was assigned 100 points, and the remaining factors were scored based on the proportion of their b value to the maximum *b* value): the academic pressure is 100 points, the CDRISC score is 30 points, and the FACESCV score is 28 points. The total score ranges from 28 to 158 points, corresponding to a risk rate range of 0.05 to 0.54. The higher the total score, the greater the risk of depression and anxiety among adolescent students (see Figure 3).

### 4.6 Validation of the predictive nomogram model and evaluation of its predictive efficacy

**Figure 4:**
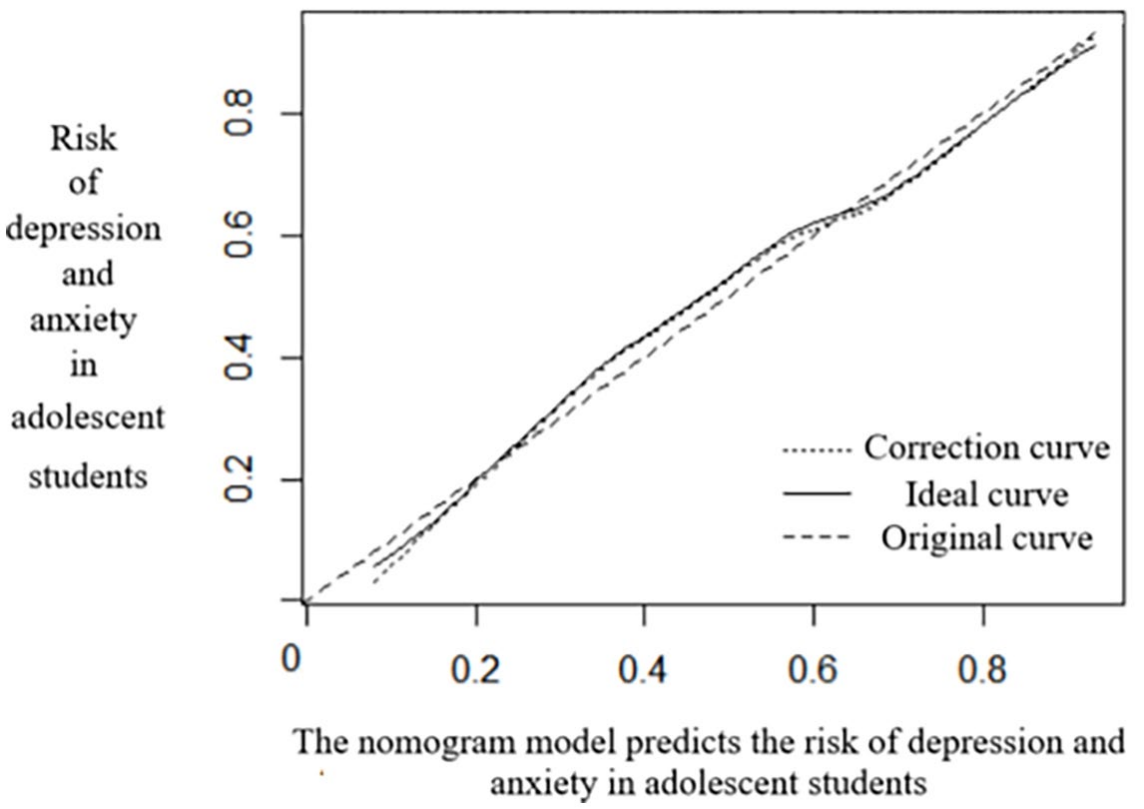
Verification curve of the predicted nomogram model.

The nomogram model was internally verified by the Bootstrap method, showing that the C-index index was 0.788 (95%CI, 0.681–0.868), and the model had good discrimination. The Calibration curve was tested for goodness-of-fit by Hosmer–Lemeshow, and the difference was not statistically significant (*χ*^2^ = 4.579, *p* = 0.321), indicating a good fit. The results of the calibration graph show that the correction curve approaches the ideal curve (*χ*^2^ = 0.264) (see Figure 4).

**Figure 5:**
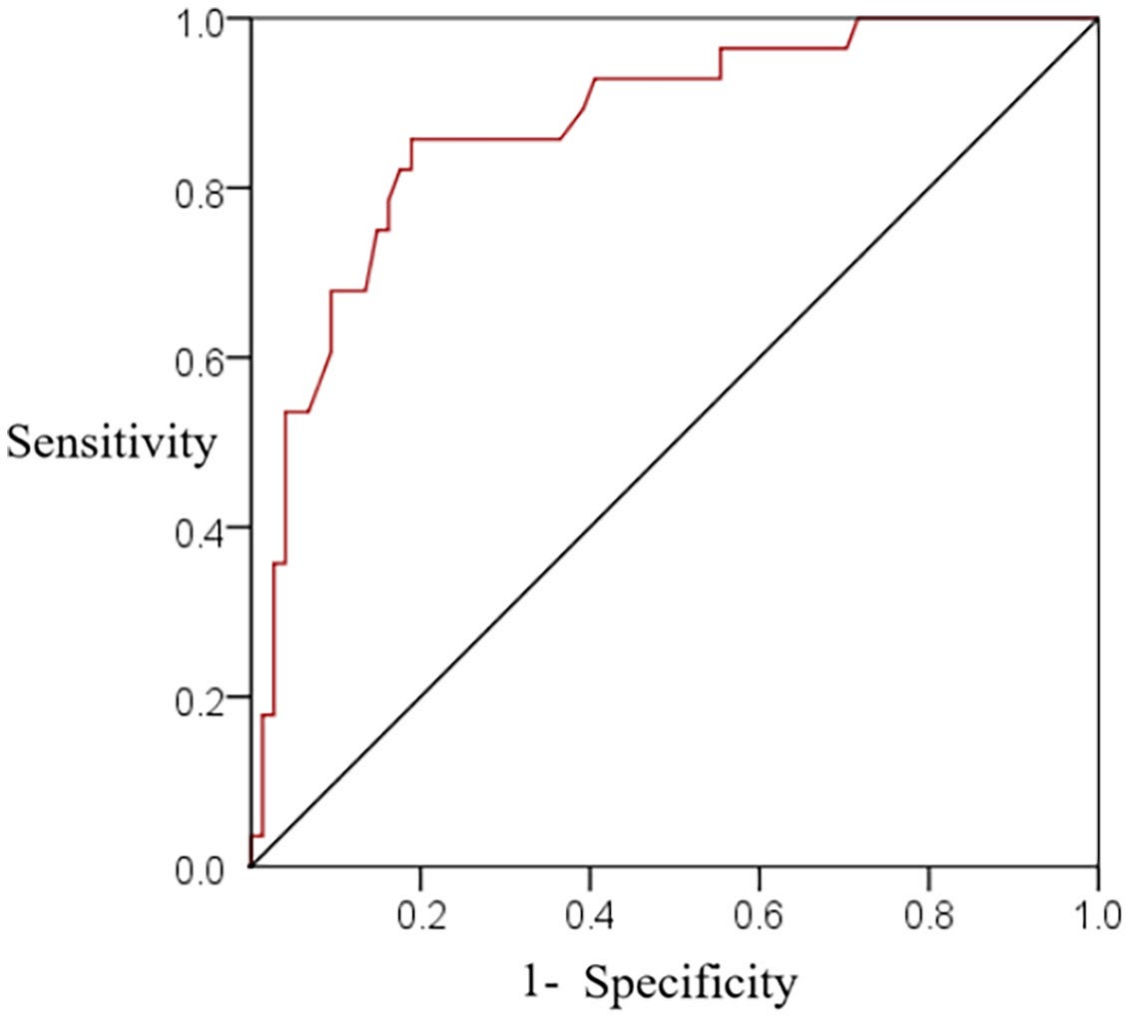
ROC curves of the training set nomogram model predicting the occurrence of depressive anxiety in adolescent students.

The nomogram model was used to predict the risk of depression and anxiety in adolescent students, the total risk was the independent variable, and the ROC curve showed that the sensitivity of the nomogram model was 80.25%, the specificity was 80.09%, and the AUC was 0.849 (see Figure 5).

**Figure 6:**
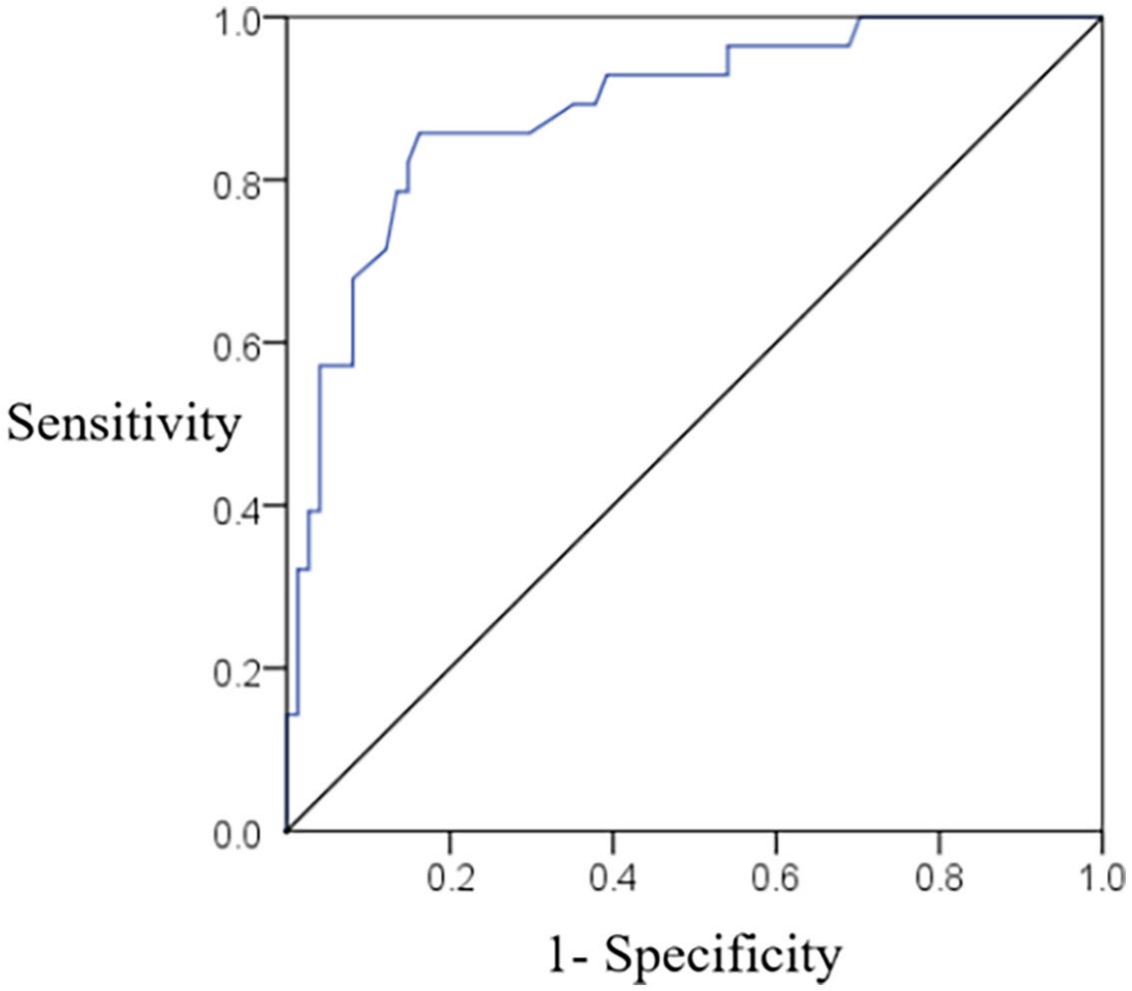
ROC curves of the validation set nomogram model predicting the occurrence of depressive anxiety in adolescent students.

The ROC curve results of the validation set showed that the nomogram model predicted the sensitivity of depression and anxiety in adolescent students with 78.23%, specificity of 82.17%, and AUC of 0.829 (95% CI, 0.727 ~ 0.922), and the prediction efficiency of the model was good (see Figure 6).

## 5 Discussion

Teenage students, as the focus of social attention, have a unique stage of physical and psychological development that is full of challenges. During this period, teenage students not only have to deal with heavy academic pressure but also handle complex and ever-changing life and social issues, all of which can lead to anxiety (34, 35). The healthy growth of adolescent students encompasses the maturation of physiological functions, the deepening of individual self-awareness, and the establishment of social relationships. At every age, attention should be paid to the construction of mental health. If growth issues are neglected, it will affect the formation of correct values in the future. The adolescent stage is often accompanied by the ambiguity of self-cognition, coupled with the relative lack of social life experience and the lack of life goals, making it more likely for adolescent students to enter the abyss of depression and anxiety in the long-term psychological distress, and thus damage the healthy development of body and mind. In the absence of appropriate guidance, supervision and psychological support, adolescent students are more likely to have bad behavior, which will exacerbate the risk of depression and anxiety symptoms (36). As an important environmental factor for the growth of adolescents, the composition and atmosphere of family structure have a profound impact on the mental health status of adolescents (37). According to relevant studies (38, 39), the positive role and interaction mode of parents and grandparents in the family can significantly affect the level of depression and anxiety among adolescent students, emphasizing the key role of family environment in the maintenance of adolescent mental health. Therefore, for the special group of young students, all sectors of society should give more attention and support, through strengthening mental health education, optimize the family environment, provide personalized guidance and other ways to help young students better cope with the challenges in growth, promote the healthy development of body and mind.

This study showed that among 920 adolescent students, 164 cases experienced depression and anxiety, the incidence was 17.83%, 756 cases had no depression and anxiety; the incidence of depression and anxiety among adolescent students in this study was higher than that of Wu et al. (40) study, the reasons for the differences are related to factors such as sample sources and regional differences. Logistic Regression analysis showed that academic stress, FACESCV and CDRISC score were the factors affecting the occurrence of depression and anxiety among adolescent students in Guilin. The study of Chen et al. (41) shows that psychological stress and psychological trauma, as the main causes of depression and anxiety symptoms in contemporary adolescent students, have become increasingly the focus of the field of mental health research. With the intensification of academic pressure, young students are under unprecedented psychological pressure, stress widespread and diverse, including but not limited to test anxiety, high expectations of family and peer relationship conflict and pressure, adolescent students long-term accumulation and if failed to get the appropriate way, easy to induce depression. In terms of psychological trauma, such as sudden changes in family structure, bullying in the campus environment and major changes in personal life, they all pose a serious threat to the mental health of young students. Traumatic experiences not only erode the basis of security and trust among adolescent students, but also weaken their ability to exercise emotional self-regulation, thus significantly increasing the risk of depression and anxiety symptoms (42). Inter-individual variation in coping with academic stress was significant. For some adolescents, moderate academic stress can be transformed into positive learning motivation and promote their academic achievement; however, excessive academic stress may become a psychological burden and have a significant negative impact on their mental health, manifested in the aggravation of emotional problems such as depression and anxiety. Therefore, it is of great significance to deeply explore the causes, influence mechanism and effective intervention strategies of psychological stress and psychological trauma to promote the mental health development of adolescents (43). The academic circle should devote itself to building a more comprehensive and scientific mental health evaluation system, and strengthen mental health education and counseling inquiry service to provide timely and effective psychological support and intervention for adolescent students (44). Zhao et al. (45) showed that high school students’ academic pressure is closely related to their own anxiety and depression. Young students are more resilient to cope with adversity and challenges, thus reducing the possibility of feeling lonely and helpless; young students with depression and anxiety are more difficult to adjust their emotions and less resilient. Rengasamy et al. (46) study also showed that psychological resilience training can improve depressive mood in adolescent students. Studies have shown that (47), resilience CDRISC scores are associated with depression and anxiety symptoms. Studies have shown that (48), the psychological resilience of adolescent students, as assessed by CDRISC, shows a significant negative correlation with its early depressive symptoms. As an internal resource, psychological resilience plays an important role in the development of resisting depressive emotions. Young students with high psychological resilience can more effectively mobilize their own resources and maintain emotional stability when facing life challenges, thus reducing the emergence of depressive symptoms. As an important external support system for the mental health of adolescent students, its good functioning has a significant role in promoting psychological resilience and reducing the risk of depression (49).

In this study, the prediction model of depression and anxiety in Guilin city was established. The ROC curve results of the validation set showed that the sensitivity of the nomogram model was 78.23%, the specificity was 82.17%, and the AUC was 0.829 (95% CI: 0.727 ~ 0.922), indicating that the model had good efficacy in predicting the occurrence of depression and anxiety in adolescent students. The nomogram model established in this study provides a new method for clinical warning of the risk of depression and anxiety among adolescent students, and its indicated risk indicators can have certain value for rapid screening of high-risk groups.

In conclusion, adolescent students have a high risk of depression and anxiety, and academic stress, FACESCV and CDRISC score are the factors affecting the occurrence of depression and anxiety among adolescent students in Guilin. The early warning model constructed based on this predicts that the occurrence of depression and anxiety among adolescent students can be good. As a single-center study, this research has a relatively limited sample size, and the singularity of the sample source may lead to certain limitations in the extrapolation of the research results, making it impossible to comprehensively reflect the current status of depression and anxiety among all adolescent students. Future research should focus on expanding the sample size and conducting multi-center collaboration to enhance the universality of the model and the accuracy of early warning, thereby more effectively guiding the prevention and intervention of depression and anxiety among adolescents.

## Data Availability

The original contributions presented in the study are included in the article/supplementary material, further inquiries can be directed to the corresponding authors.

## References

[1] Zhang CC Wang YQ. Education burden reduction, family education investment and education inequality. Manag World. (2022) 38:83–97. doi: 10.19744/j.cnki.11-1235/f.2022.0134

[2] Yan Q Liu Y. Social support for depression groups-based on the survey of “depression”. Press. (2022) 6:45–56. doi: 10.13479/j.cnki.jip.2024.03.034

[3] Almadani HA Alsubaihi AA Alsqabi AH Mohammed AA Meshal IA Osama AA . Comparison of depression and anxiety in first- versus non-first generation Saudi medical students: a cross-sectional study. Medicine. (2024) 103:39115. doi: 10.1097/MD.000000000039115 PMC11272298 39058812

[4] Huang XX Li YT Chen JH Ma JJ Cong EZ Xu YF. Effect of family structure on symptoms of depression and anxiety in adolescents: the mediating role of affective neglect. Chin J Contemp Pediatr. (2023) 25:80–5. doi: 10.7499/j.issn.1008-8830.2208058 PMC9893832 36655668

[5] Liu LL Hu JT Cao Y Wang D Zhang K Zhu F.. Research on the relationship between coping style and anxiety and stress perception in adolescents with non-suicidal self-injury behavior. J Psychiatry. (2023) 36:349–52. doi: 10.3969/j.issn.2095-9346.2023.04.003

[6] Dunn EC Gilman SE Willett JB. The impact of exposure to interpersonal violence on gender differences in adolescent-onset major depression: results from the National Comorbidity Survey Replication (NCS-R). Depress Anxiety. (2012) 29:392–9. doi: 10.1002/da.21916, 22447513 PMC4136968

[7] Yang T Gao JQ Wei NN Zhang XB Ma CX. Inner Mongolia high anxiety symptoms related factors analysis. J School Health China. (2024) 6:849–53. doi: 10.16835/j.cnki.1000-9817.2024165

[8] Lu YB Li BH Zhan NN Wen FW. Life events and depression in junior high school students: the teacher in charge of emotional labor regulation. Chin J Clin Psychol. (2014) 22:885–8. doi: 10.16128/j.cnki.1005-3611.2014.05.075

[9] Stange PJ Hamlat JE Hamilton LJ Abramson LY Alloy LB. Overgeneral autobiographical memory, emotional maltreatment, and depressive symptoms in adolescence: evidence of a cognitive vulnerability–stress interaction. J Adolesc. (2013) 4:201–8. doi: 10.1016/j.adolescence.2012.11.001 PMC3530666 23186994

[10] Luca DB Giovanni DSH Gotlib IH. A cognitive model of depression and political attitudes. Electoral Stud. (2024) 87:21–6. doi: 10.1016/J.ELECTSTUD.2023.102737

[11] Shi ZJ Ding XH Cui LJ Du J Chen MQ Long QW . Changes in functional connectivity of the prefrontal temporal lobe in adolescents with depression: a near-infrared brain imaging study. J Chongqing Med Univ. (2025) 21:1–7. doi: 10.13406/j.cnki.cyxb.003717

[12] Yu R Liu N Liao KK Chen XY Peng SJ Li Y . A study on the changes of gray matter structure in the brains of non-suicidal self-harming adolescents with major depressive disorder magnetic resonance imaging. Magnetic Resonance Imaging. (2024) 15:73–8. doi: 10.12015/issn.1674-8034.2024.12.011,

[13] Zeng ZH Hu YQ Liu SJ Peng LY Yang Q Wang HC . The influence of school interpersonal relationships and the cumulative genetic risk of polygenic serotonin system on adolescent depression. Psychol Dev Educ. (2025) 9:436–47. doi: 10.16187/j.cnki.issn1001-4918.2025.03.14

[14] Gong YH Ye YY Zhou X. Youth abuse and neglect of the influence of depression: the role of self-esteem and self-disclosure. J Psychol Dev Educ. (2024) 40:886–93. doi: 10.16187/j.cnki.issn1001-4918.2024.06.13

[15] Xin GG Zhang LB Chang RS Zhang YL. The development trajectory of bullying in early adolescence: the predictive effects of depression, self-esteem and academic achievement psychological development and education (2023) 33:568–79. doi: 10.16187/j.cnki.issn1001-4918.2023.04.13

[16] Yuan GX Zheng SX Xiong GQ Ding ZX Song Q Luo XW . The influence of family environment on depression in adolescence: a regulating intermediary model. Chin J Clin Psychol. (2023) 31:555–61. doi: 10.16128/j.cnki.1005-3611.2023.03.010

[17] Cheng QL Xie L Wang L Wu YF Huang YY Jia QJ . Analysis of influencing factors of depressive tendency in adolescents. Chin J Public Health. (2022) 38:680–5. doi: 10.11847/zgggws1136967

[18] Hu R Peng LL Jiang LH Zhao L. Adversity beliefs and social support condition's influence on adolescent depressive symptoms of Chengdu. J Med Soc. (2024) 5:67–73. doi: 10.13723/j.yxysh.2024.11.010

[19] Song LL Wu J Su PY. Environment controllable factors of adolescent depression. J Sch Health China. (2022) 2:312–5. doi: 10.16835/j.carol.carroll.nki.1000-9817.2022.02.035

[20] Li X Zhang C Yu RZ Yin Yj Zhou T Liu W . The combined effect of childhood emotional abuse and bullying victimization in the development of depressive symptoms in adolescents: sequential mediation or enhanced regulation. J Psychol. (2025) 57:1056–69. doi: 10.3724/SP.J.1041.2025.1056

[21] Lin YR Zhang XQ Chen LN Zhang YJ Sun Q Liu HQ . The mediating moderating role of social support and stressful life events between positive parenting styles of parents and depressive mood in adolescents in high-altitude areas. Chin J Child Care. (2025) 4:444–50. doi: 10.11852/zgetbjzz2024-0764

[22] Jiang XY Chen Z Zhao L Zhou JM Ning Z Wang H. Youth peer relations and depression, anxiety, cross lag analysis. Modern Prevent Med. (2025) 52:1986–91. doi: 10.20043/j.cnki.MPM.202501298

[23] Cao YM Fang HC Zhu XY Ji LQ Zhang WX. BDNF gene, peer relationship and early adolescent depression: from a dynamic development perspective. J Psychol. (2023) 10:1620–38. doi: 10.3724/SP.J.1041.2023.01620

[24] Cao CH Liao XL Gamble JH Li LL Jiang XY Li XD . Psychometric properties of the Chinese version of the depression anxiety stress scales for youth (DASS-Y-C) and its application. Curr Psychol. (2025) 15:1–15. doi: 10.1016/S0005-7967(96)00068-X

[25] Ren XB Xu HX Zhong RS Duan X Yu J. Current situation and early warning model construction and verification of depressive symptoms among adolescents from single-parent families in Jianyang City. Chin Sch Health. (2024) 16:1096–100. doi: 10.16835/j.cnki.1000-9817.2024231

[26] Chen Y Hu XX Wang S. Model based on multiple domain feature combining CBAM depression of EEG signals recognition. J Harbin Univ Sci Technol. (2024) 29:1–10. doi: 10.15938/jjhust.2024.03.001

[27] He JB Zhang YY Liao BY Deng XX Sun MJ Zhang ZY . Adolescent anxiety depression perceived stress and eating behavior of network analysis. Chin Sch Health. (2025) 17:1–6. doi: 10.16835/j.cnki.1000-9817.2025185

[28] Wei JL Ma ZJ Buwei Zorem Aili. Adolescent depression symptoms characteristic and the sleep quality and mental toughness relationship. Chin Sch Health. (2025) 18:1–5. doi: 10.16835/j.cnki.1000-9817.2025176

[29] Zhou XW Zhang XY Ma YY Chen YY Xu T Zhang TC . Analysis of the development trajectory of early depressive symptoms among adolescents in the border region of Hunan, Hubei, Chongqing and Guizhou based on the GBTM model modern preventive medicine. Magnetic Resonance Imaging. (2025) 52:1980–5. doi: 10.20043/j.carol.carroll.nki.MPM.202412517,

[30] Hudson CC Hall L Harkness KL. Prevalence of Depressive Disorders in Individuals with Autism Spectrum Disorder: a Meta-Analysis. J Abnorm Child Psychol. (2019) 47:165–75. doi: 10.1007/s10802-018-0402-1 29497980

[31] Huang AQ Peng ZL Chen J Lei W Wang X Liu KZ . The relationship between self-efficacy and insomnia among college students: the mediating role of negative emotions. J Psychiatry. (2021) 34:97–100. doi: 10.3969/j.issn.2095-9346.2021.02.001

[32] Yu X Zhang J. Factor analysis and psychometric evaluation of the Connor-Davidson resilience scale (DASS-21) with Chinese people. Soc Behav Pers. (2007) 35:19–30. doi: 10.2224/sbp.2007.35.1.19

[33] Sun ZX Liu HX Jiao LY Zhou T Yang LN Fan JY . Reliability and validity study of hospital anxiety and depression scale. Chin J Clin Phys. (2017) 11:198–201. doi: 10.3877/cma.j.issn.1674-0785.2017.02.005

[34] Tian LF Huang Q. An exploratory study on the intimacy and adaptability of the family. Chin J Health Psychol. (2009) 17:869–72. doi: 10.18502/ijps.v19i1.14338

[35] Zhao MY Xu SJ Li XH Xu HQ Chen DY Zhu Y . Association of vitamin D deficiency with anxiety and depressive symptoms among middle school students in Shenzhen. Sch Health China. (2023) 44:1030–3. doi: 10.16835/j.cnki.1000-9817.2023.07.016

[36] Wang Y Peng C Cheng JH Rong FJ Hu J Xu ZX . The valence of suicide behavior and its association with depression and anxiety in 5 provinces of China. Public Health China. (2023) 39:1225–31. doi: 10.11847/zgggws1141897

[37] Xie Q Ge MQ Li H Xu JL Song YJ Su F . Analysis of factors associated to depressive anxiety symptoms and physical activity in rural reflux adolescents. School Health China. (2023) 44:1038–43. doi: 10.16835/j.cnki.1000-9817.2023.07.018

[38] Jiang S Ding JQ Liu Y Lu YY Li XQ Chen J . Impact of cyberbullying / bullying on sleep quality in early adolescence: chain mediators of social anxiety and depressive mood. Psychol Dev Educ. (2023) 39:85–96. doi: 10.16187/j.cnki.issn1001-4918.2023.01.10

[39] Deng LY Wang YQ Yang YM Zhou N Li BL . Parental anxiety/depression and adolescent electronics addiction such as mobile phones during the epidemic: a chain mediation model. Chin J Clin Psychol. (2021) 29:1230–6. doi: 10.16128/j.cnki.1005-3611.2021.06.022

[40] Zhang YH Li JY Yin XQ Wang JL. Relative weight analysis of the relationship between types of childhood abuse and anxiety and depression among adolescents. Sch Health China. (2022) 43:407–10. doi: 10.16835/j.cnki.1000-9817.2022.03.021

[41] Wu DY Wang MF Zhang B Lai WY Gu D. Changes in depression and anxiety status of middle school students at the beginning and the end of 2021 in Chongqing. Sch Health China. (2022) 43:736–8. doi: 10.16835/j.cnki.1000-9817.2022.05.022

[42] Chen JY Zhao Y Li KH Tang CM Yan MX Zhang Q . The current situation of cyberbullying in children and adolescents and its association with anxiety and depression symptoms. Modern Prevent Med. (2022) 49:808–13. doi: 10.19818/j.cnki.issn.1001-9553.2022.05.009

[43] Gu WX Tan YL Lu WY Du LDD Zhu JF. Current situation of eating behavior and the influence of depression and anxiety among adolescents in Shanghai. Sch Health China. (2022) 43:864–8. doi: 10.16835/j.cnki.1000-9817.2022.06.016

[44] She WB Zhang QQ. House of conflict: social support and digital stress in the online depression community. News Reporter. (2022) 10:85–96. doi: 10.16835/j.cnki.1000-9817.2024114

[45] Liu FD Yang YM Xiao Y Jiang XL Wang XY Sheng JT . The relationship between anxiety, insomnia and family intimacy and internet addiction and non-suicidal self-injury behaviors in middle school students. School Health China (2023)44: 1770–1774.doi:10.16835/j.cnki.1000-9817.2023.12.003

[46] Zhao Ke Yin SQ Liu H. The influence of bullying, bullying tolerance and cognitive emotion regulation strategies on anxiety and depression in children and adolescents. Chin J Child Health Care. (2024) 32:268–272, 279. doi: 10.11852/zgetbjzz2023-1046

[47] Rengasamy M Marsland A McClain L Kovats T Walko T Pan L . Longitudinal relationships of cytokines, depression and anhedonia in depressed adolescents. Brain Behav Immun. (2021) 91:74–80. doi: 10.1016/j.bbi.2020.09.004, 32919038 PMC7952030

[48] Jung SJ Jeon YJ Choi KW Yang JS Chae JH Koenen KC . Correlates of psychological resilience and risk: prospective associations of self-reported and relative resilience with Connor-Davidson resilience scale, heart rate variability, and mental health indices. Brain Behav. (2021) 11:e02091. doi: 10.1002/brb3.2091, 33638932 PMC8119814

[49] He PY Chen YW. Parental marital conflict and early depression and loneliness among adolescent students: the chain mediation role of parental psychological control and psychological resilience among adolescent students. Chin J Behav Med Brain Sci. (2023) 32:1018–24. doi: 10.16835/j.cnki.1000-9817.2023.11.014

